# Retraction: Green Synthesis of Metallic Nanoparticles and Their Potential Applications to Treat Cancer

**DOI:** 10.3389/fchem.2022.1133173

**Published:** 2023-01-12

**Authors:** Frontiers Editorial Office

Following publication, we received concerns that images published in the article had been appropriated without permission from a separate research group. An investigation was conducted in accordance with Frontiers’ policies that confirmed this. The authors and their institutions remained unresponsive to our multiple attempts to contact them. Therefore, the article has been retracted.

The authors have not responded to correspondence regarding this retraction.

This retraction was approved by the Chief Editors of Frontiers in Chemistry and the Chief Executive Editor of Frontiers.

